# Simpler and effective radiological evaluations for modiolar proximity of a slim modiolar cochlear implant electrode

**DOI:** 10.1038/s41598-020-74738-x

**Published:** 2020-10-19

**Authors:** Sang-Yeon Lee, Jin Hee Han, Marge Carandang, Yun Jung Bae, Byung Yoon Choi

**Affiliations:** 1 Department of Otorhinolaryngology-Head and Neck Surgery, Seoul National University Bundang Hospital, Seoul National University College of Medicine, 300 Gumi-dong, Bundang-gu, Seongnam, 463-707 Republic of Korea; 2 Department of Otorhinolaryngology-Head and Neck Surgery, East Avenue Medical Center, Metro Manila, Philippines; 3 Department of Radiology, Seoul National University Bundang Hospital, Seoul National University College of Medicine, 300 Gumi-dong, Bundang-gu, Seongnam, 463-707 Republic of Korea

**Keywords:** Anatomy, Diseases, Medical research

## Abstract

A new slim modiolar electrode (CI532/632) has been reported to ensure better modiolar proximity than conventional electrodes. Better modiolar proximity has been proposed to yield better electrode discrimination capability and potentially better speech outcomes, necessitating its efficient measurement. Currently, intracochlear positional index (ICPI), the most reliable indicator for evaluating modiolar proximity, has been measured exclusively through ‘metal artifact-less’ cone beam CT. However, popular use of this index is precluded due to lack of cone beam CT in many institutions. Thus, eyes are now on elucidation of easy-to-measure indicators of modiolar proximity derived from conventional CT, which is accessible in all centers. We observed that enhanced tomographic resolution significantly reduces partial volume artifacts, providing better visualization of modiolus-electrode distance. Aided by ultra-high kernel specification with high-resolution index, we developed a novel and easy-to-measure, conventional CT-specific indicator, “modified ICPI”, for evaluation of modiolar proximity. Further, we showed that it closely correlates with the previously proposed parameter of modiolar proximity, the spiral diameter, measured from post-insertion radiograph, reiterating the value of X-ray-based spiral diameter. Through this study, we have taken a step toward the stage of immediate visual feedback regarding modiolar proximity and changes in insertion technique intraoperatively, ensuring optimal modiolar proximity.

## Introduction

The new slim modiolar electrodes (e.g., CI532 or CI632) combine the slim electrode diameter of slim-straight electrodes and the modiolus-hugging feature of conventional perimodiolar electrodes. This new commercially available slim modiolar electrode ensures better modiolar proximity^1,2^ and provides substantial preservation of residual hearing^3–11^. For modiolar hugging electrodes, better auditory performance depends on the final intracochlear positioning of the electrode array, such as scalar location and modiolar proximity^12,13^. The slim modiolar electrodes have been reported to provide a consistently higher scalar tympani position than conventional perimodiolar electrodes^6,14^. In addition, 4.1–4.6% rate of tip-fold over has been reported in the literature^9,14^, but tend to gradually decrease with experience^15^. Thus, specifically for slim modiolar electrodes, degree of modiolar proximity may be one of the critical determinants of better auditory performance. Recently, cochlear implant recipients with slim modiolar electrodes (i.e., CI532/632) led to either similar or statistically superior audiological results compared with those with straight electrodes (i.e., CI422/522) strictly matched for age and preoperative hearing thresholds^2^. Furthermore, a growing body of evidence suggests that the enhanced modiolar proximity of slim modiolar electrodes would lead to improved place-pitch spectral discrimination^16^, and speech perception outcomes^14,17^, as compared with conventional perimodiolar electrodes. However, not all reports in literature fully support this phenomenon^9,18,19^, probably due to confounding variables such as heterogeneous degrees of modiolar proximity even with slim modiolar electrodes^20^. Indeed, better modiolar proximity has been proposed to attenuate the substantial overlap (i.e., spread of excitation) in the electrical field between electrode contacts, resulting in better electrode discrimination capability^16,21^, and potentially better speech outcomes than conventional electrodes^21^. In addition, positioning of the electrodes close to the spiral ganglion neurons can reduce the effects of neural-electrode interaction by improving channel discrimination^16^, lowering threshold levels^22^, and reduction of power consumption^23^. Therefore, robust and efficient measurement of modiolar proximity is mandatory for slim modiolar electrodes which were designed to achieve the best modiolar proximity amongst all electrodes.

To date, there are different approaches to measure the electrode position relative to the modiolar wall distance. One of the widely accepted measurements is the wrapping factor to determine how the electrode array is located relative to the lateral wall^24^. The wrapping factor has been suggested to be inversely correlated with the degree of modiolar proximity. However, the wrapping factor has been reported to yield an inconsistency to determine the relative relationship of the electrode array against the lateral wall between the cone-beam CT and the histologic finding, requiring a more accurate and refined radiological value for modiolar proximity^25^. Recently, intracochlear positioning of the electrode array in close proximity to the modiolus—with good modiolar hugging, as evidenced by the wrapping factor normalized or intracochlear positioning index (ICPI)^25^—would lead to better hearing outcomes for cochlear implantees with the slim modiolar electrodes.

With the advent of cone-beam CT based on flat-panel volumetric technology, significantly better image quality enables clear delineation of the intracochlear electrode array with more definition of the fine osseous structures surrounding the temporal bone^26,27^. Although a previous ex vivo study showed that the average electrode-to-modiolus distance value measured by two imaging modalities (conventional CT vs. cone-beam CT) was not different^28^, measurement of electrode-to-modiolus distance using conventional CT is not feasible due to metal artifacts blurring the region of interest. A recent study proposed the most reliable and finely tuned indicator reflecting the degree of modiolar proximity, called ICPI^25^, which was obtained exclusively through ‘metal artifact-less’ cone-beam CT. However, lack of such cone-beam CT in many institutions preclude widespread use of this index. Thus, eyes are now on the elucidation of easy-to-measure indicators of modiolar proximity derived from conventional CT that is accessible to all cochlear implantation centers.

Herein, we observed that higher tomographic resolution significantly attenuates imaging blurring and enhances imaging sharpness, thereby enabling elaborate measurement of electrode-to-modiolus distance even in conventional CT. Aided by ultra-high kernel specification with a high-resolution index, we came up with a novel and easy-to-measure, conventional CT-specific indicator, modified ICPI, for evaluation of modiolar proximity. Further, we evaluated whether or not modified ICPI obtained from conventional CT correlates with the previously proposed parameter of modiolar proximity, the spiral diameter, measured from a post-insertion radiograph during or after the operation. If that is the case, simple X-ray would eventually be a nice substitute for conventional CT in terms of obtaining modiolar proximity and comparison of it just for practice within a single center. This should be the case especially for pediatric patients, where radiation matters. However, the role of ICPI obtained from conventional CT does not seem to swiftly wane, especially for comparison of the parameter across centers, especially for adults. Simple X-ray technique is not standardized across centers, precluding comparison of modiolar proximity values across centers. Therefore, CT measurement should not be underestimated as just an interim approach, but it would better regard the use of CT as a valuable independent approach per se. Collectively, these results offer otologists a novel and easy-to-calculate indicator for modiolar proximity through conventional CT and reiterates the value of X-ray-based measurement of the spiral diameter, enabling immediate visual feedback and changes in insertion technique intraoperatively, thereby ensuring optimal modiolar proximity.

## Methods

### Participants

We retrospectively reviewed cochlear implant recipients in whom slim modiolar electrodes, such as the CI532 or CI632, were implanted by a single surgeon (B.Y.C) using the round window approach, exclusively with the pull-back technique. Recently, the insertion technique that ensures better modiolar proximity, called the pull-back maneuver, has been introduced for slim modiolar electrodes, based on a human cadaveric temporal bone study^29^. Only patients with 0.4 mm slice thickness on temporal bone CT and a high-resolution filter specification were included. Subjects with the following conditions were also excluded from this study: (1) history of explantation or reimplantation, (2) severe cochlear ossification, and (3) obvious cochlear anomalies on radiological images based on the classification of inner ear anomalies. Ultimately, a total of 30 recipients (33 ears) of CI532 (N = 15, 16 ears) or CI632 (N = 16, 17 ears) cochlear implants were enrolled. In our current study, two of 33 ears were implanted using the extended pull-back maneuver^20^, due to short cochlear duct length measured preoperatively. The study protocol and a waiver of consent for this retrospective chart review were approved by the review board of the Clinical Research Institute at Seoul National Bundang Hospital (approval no. IRB-B-2004/604-119). All methods employed in this study were in accordance with the approved guidelines and the Declaration of Helsinki.

### CT protocol

High-resolution temporal bone CT scans were performed on the day after the surgery, particularly in adult cochlear implant recipients, whenever available. Axial images were obtained with 0.4 mm slice thickness using 120 kV, 64 × 0.6 mm collimation, 1-s rotation, pitch factor of 0.85, and 205 mAs, in accordance with the age of the subjects by 256-channel multi-detector computed tomography (SOMATOM Force, Siemens Healthineers, Forchheim, Germany)^30^. All data were reconstructed using two sets of ultra-high resolution kernels with different resolution indices (Uh59, resolution index of 8.3 line-pair (lp)/cm; Ur81, 19.7 lp/cm). Importantly, no difference in effective dose was found between the two different filters (Uh59 vs. Ur81), as they are based on the difference in the reconstruction algorithm of the raw data obtained from the imaging scans, regardless of the scan parameters such as kVp and mAs in the conventional temporal bone CT.

### Quantitative and qualitative assessments

**Figure 1:**
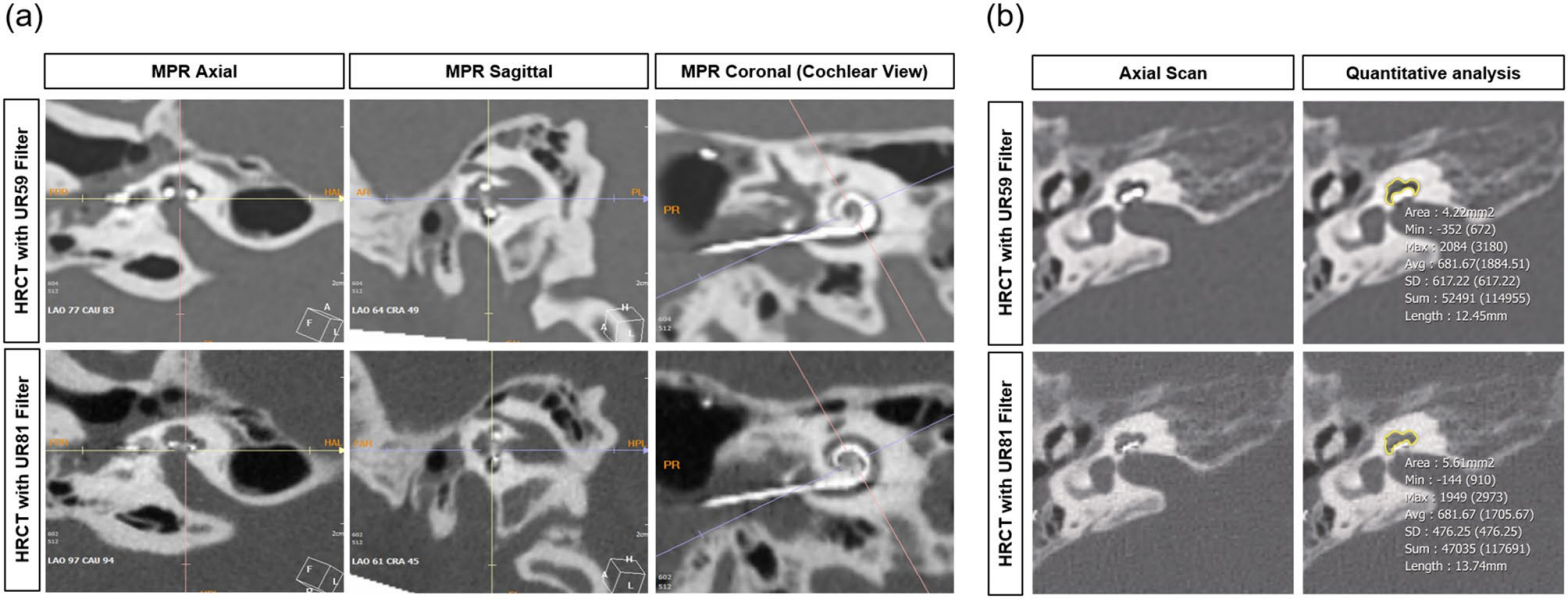
Reformatted conventional computed tomography along the plane of the “Cochlear View” by a multiplanar reconstruction algorithm. (**a**) Based on the “Cochlear View”, qualitative assessments of the slim modiolar electrode array were compared between the two resolution indices of UR59 (low resolution index, upper panel) and UR81 (high resolution index, low panel) in terms of imaging sharpness and overall subjective image quality. (**b**) For quantitative assessments, the noise level of the region of interest (yellow line) obtained by the middle turn of the cochlea was compared between conventional computed tomography with two different tomographic resolutions using ultra-high kernel specification. HRCT, high-resolution computed tomography; MPR, multiplanar reconstruction.

As shown in Fig. 1a, high-resolution temporal bone CT images were reformatted to create the “Cochlear View,” following multiplanar reconstruction (MPR) algorithm^31,32^. A board-certified neuroradiologist (Y.J.B, with 10 years of experience) and an otologic surgeon (B.Y.C) independently performed visual inspection of the “Cochlear View” on the conventional CT with ultra-high kernel specification of Uh59 (low resolution index) and Ur81 (high resolution index). As modified from the version of Kidoh et al.^33^, the degree of image sharpness under the bone window setting (window level and width of 300 and 4000 Hounsfield units, respectively,) was assessed using a 4-point visual score, and the overall subjective image quality was separately examined according to a 5-point visual score (Table 1). The inter-class correlation was excellent with regard to imaging sharpness and overall subjective image qualities (Table S1).

**Table 1:** Definition of visual scores for the qualitative analysis.

Imaging sharpness		Overall subjective image quality	
Score	Definition	Score	Definition
1	Marked blurring of electrode without definable margins relative to cochlear lateral wall	1	Severe artifact, non-diagnosable image
2	Moderate Blurring, but with definable margins	2	Poor image quality, partially non-diagnosable
3	Minimal blurring	3	Moderate image quality, limited diagnostic confidence
4	Sharp definition	4	Good image quality, sufficient for diagnosis
		5	Excellent image quality with no artifact

As shown in Fig. 1b, a neuroradiologist (Y.J.B) obtained an axial scan to compare the noise level between the two different resolution indexes. The reader allocated the region of interest (ROI) in the slice where the middle turn of the cochlea was clearly visible. The noise level was defined as the standard deviation of the Hounsfield units in each ROI of the structures. Values were measured twice on the same image, and the average of the two values was used for further analysis.

### Intracochlear position index

**Figure 2:**
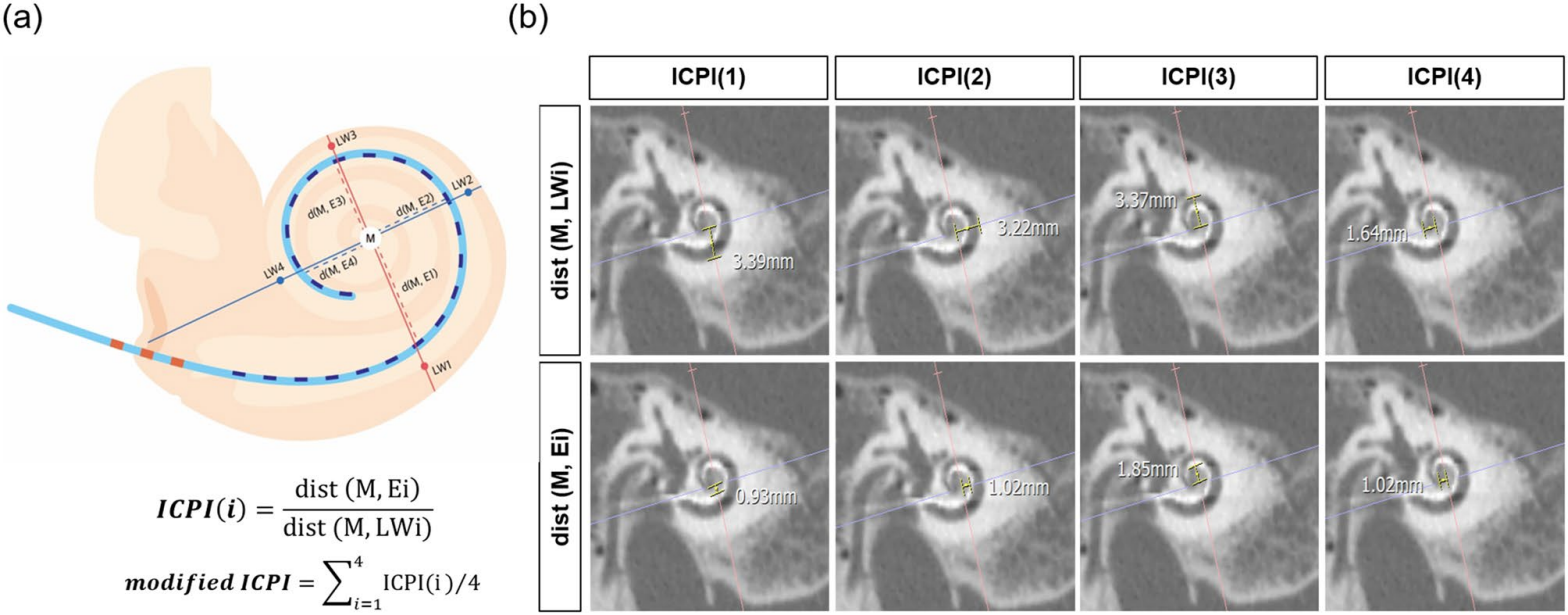
A novel conventional CT-specific indicator for evaluation of modiolar proximity. (**a**) A schematic illustration explaining the modified intracochlear position index (ICPI) measurements technique. (**b**) Under two-axes’ cross modiolus on “Cochlear View”, the metrics reflecting modified intracochlear position index (ICPI) at four fixed points were measured on conventional computed tomography using ultra-high kernel specification with high-resolution index. These values were measured at fixed positions based on the two lines (red and blue) associated with the modiolus and the round window. *M* modiolus, *E* electrode contact, *LW* lateral wall, *dist* distance.

With reference to a recent study by Miguel et al.^25^, ICPI(i) refers to the ratio of the Euclidean distance between the modiolus (M) and the electrode (Ei) relative to the distance between the modiolus and the lateral wall (LWi). Specifically, the modiolus (M) value was determined as a crossing point in relation to the two lines associated with the modiolus and the round window. In this study, we defined the “modified ICPI,” a novel parameter that reflects the degree of modiolar proximity. The modified ICPI is the average value of ICPI(i) measured at four fixed positions under a two-axis crossing modiolus (Fig. 2a). As depicted in Fig. 2b, ICPI(i) and modified ICPI on the Cochlear view were measured to the nearest 0.01 mm by a neuroradiologist (Y.J.B) blinded to all subject-related information using software on the PACS workstation combined with sharpening and zooming (Infinitt, Seoul, South Korea). Values of ICPI(i) were computed twice on the same image, and the average of the two values was used for further analysis. Also, the angular depth of insertion was measured as a reference for the angle between the round window and electrode tip on the “Cochlear view” reformatted by the multiplanar reconstruction algorithm^31^.

$$\begin{aligned}&{ICPI}\,\left({{i}}\right)=\frac{\mathrm{dist }\left(\mathrm{M},\mathrm{ Ei}\right)}{\mathrm{dist }(\mathrm{M},\mathrm{ LWi})}\\ &{{modified}}\,{{ICPI}}=\sum\limits _{i=1}^{4}\mathrm{ICPI}(\mathrm{i})/4.\end{aligned}$$

### Spiral diameter

**Figure 3:**
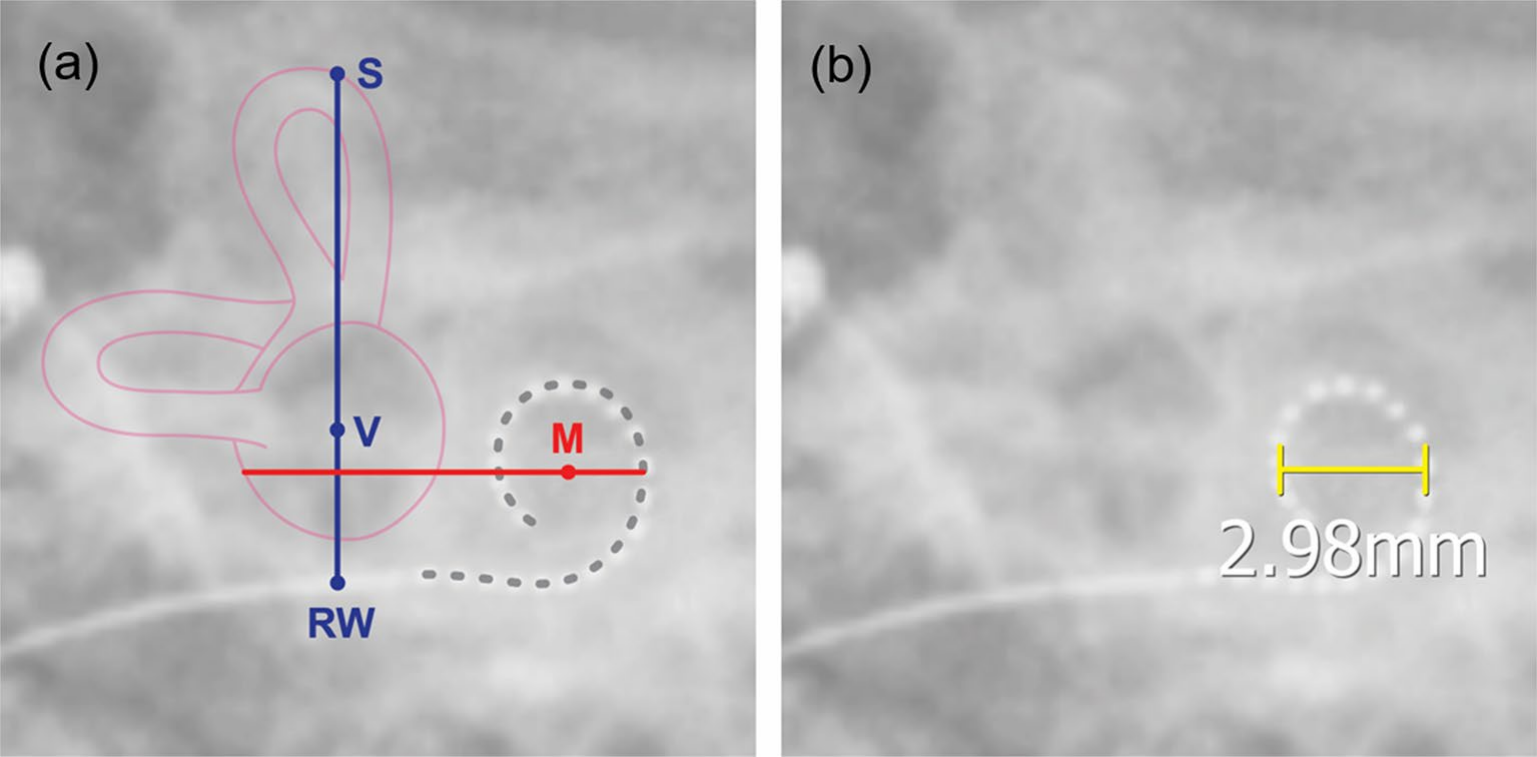
Landmark -based measurement of a spiral diameter on the postinsertion radiography. (**a**) The spiral diameter of the spiral configuration of the electrode array was measured on a horizontal line (red line) across the modiolus (M). This line is positioned vertically based on a reference line (blue line) connecting the apex of the superior semicircular canal (S) to the vestibule (V) and the round window (RW). (**b**) A representative X-ray-based spiral diameter on an unmarked radiograph of a right cochlea.

The spiral diameter was measured from transorbital X-ray images taken the day after implantation. As illustrated in Fig. 3a, the spiral diameter of the electrode turn (Fig. 3b) was defined as the distance of the electrode turn measured on a horizontal line across the modiolus.

### Intraoperative electrically evoked compound action potential thresholds

After the final intracochlear positioning of the electrode arrays, telemetry recordings were made under sterile conditions in the operating field. “Electrically evoked compound action potential (ECAP) thresholds” were measured in every channel for all subjects using software-based neural response telemetry (NRT) recordings (Cochlear Custom Sound 4.0) with automatic NRT mode. As outlined by guideline, stimulation rate (Hz), maximum current level (CL), and the number of sweeps were set as 250 Hz, 255 CL, and 35, respectively. Furthermore, to evaluate and describe the scalar position of the electrode arrays, ‘NRT ratio’ was proposed previously^34,35^. The NRT ratio was obtained by dividing the average NRT value from electrodes 18 to 16 in the apical regions by the average NRT value from electrodes 8 to 6 in the basal regions of the electrode array^34,35^. Specifically, values > 1.05 (i.e., cut-off value) indicate scalar translocation of the inserted electrodes^34,35^.

### Statistical analyses

All statistical analyses were performed using R Statistical Software (R version 3.5.2: Foundation for Statistical Computing, Vienna, Austria) and RStudio (RStudio-1.2.5042, https://www.rstudio.com/). Then, all the analyses illustrated used the GraphPad Prism version 8.0.0 for Windows, GraphPad Software, San Diego, California USA (www.graphpad.com), An independent t-test (two-tailed) was used to compare the quantitative and qualitative assessments between the two different filter specifications (UR59 versus UR81). One-way ANOVA (within-subject design) and Tukey’s post-hoc test was used to determine if the ICPI(i) value at the four points differ from each other. Pearson correlation analyses were performed to identify the relationships between the modified ICPI, related parameters, and spiral diameters, because these parameters were normally distributed. *P* values < 0.05 were considered statistically significant.

## Results

### Demographic and clinical characteristics

The demographic and clinical characteristics of the patients are shown in Table 2. All patients were adults, and the mean age at implantation was 56.7 ± 19.5 years (range 18–91 years). No subject in this study exhibited inner ear anomalies based on temporal bone CT scan and/or internal acoustic canal magnetic resonance imaging (MRI). Twenty subjects (66.7%) had idiopathic progressive sensorineural hearing loss. With regard to the definite etiology of the deafness, the causative variants were observed in eight (26.7%) (Table S1), followed by otosclerosis (N = 1, 3.3%) and advanced chronic otitis media (N = 1,3.3%). Based on the high-resolution CT, we ensured the scalar tympani localization of the electrode array in all cases. Consistent with this, the NRT ratios of all patients measured three months after cochlear implantation showed less than the cut-off value (i.e., 1.05), strongly suggesting that the slim modiolar electrode array was placed within the scala tympani as previously suggested^34,35^. Furthermore, tip rollover of the electrode array was not observed in our cohort.

**Table 2:** Demographics and clinical characteristics.

	Cohort (N = 30, 33 ears)
**Age at CI**	
Mean [SD]	56.7 [SD: 19.5]
Range	18–91
**Sex**	
Male	16 (53.3%)
Female	14 (46.7%)
**Laterality**	
Right	20 (60.6%)
Left	13 (39.4%)
**Electrode**	
CI532	16 (48.5%)
CI632	17 (51.5%)
**Approach**	
RW/PB	31 (93.9%)
RW/extended PB^a^	2 (6.1%)
**Etiology**	
Genetic variants^b^	8 (26.7%)
Otosclerosis	1 (3.3%)
Chronic otitis media	1 (3.3%)
Idiopathic, progressive	20 (66.7%)

*CI* cochlear implantation, *SD* standard deviation, *RW* round window, *PB* pull-back technique.

^a^Note that two cases with shorter cochlear duct length underwent the extended pull-back approach to obtain better modiolar proximity of CI532/CI632.

^b^Note that genotype profile on causative variants is described in Supplementary Table S1.

### Qualitative and quantitative image analysis

The average visual scores for imaging sharpness and subjective image quality were significantly higher in the conventional CT with a high-resolution filter than with a standard-resolution filter (*P* < 0.001 by independent t-test) (Fig. S1). The size of the ROI was fixed as 6.00 ± 2.36 mm^2^ for the cochlea. The noise level was significantly lower with conventional CT with a high-resolution filter specification than with a standard-resolution filter (*P* < 0.001 by independent t-test) (Fig. S2).

### Definition and characterization of modified intracochlear positional index (modified ICPI)

**Figure 4:**
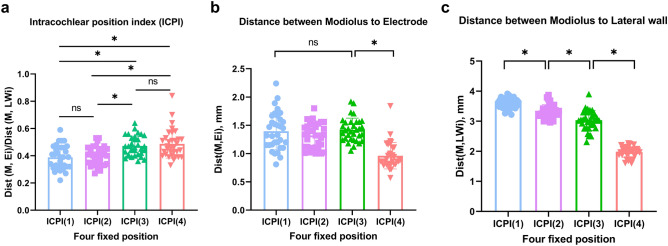
Spatial information on the intracochlear positioning of slim modiolar electrode, which is primarily related to the distance of each contact relative to the modiolus at specific points, implanted with a pull-back technique via a round window approach. (**a**) Comparison of modified intracochlear position index (ICPI)-related metrics at four points. (**b**) Comparison of the distance from the modiolus to the electrode contact (i.e., Dist(M,Ei)) at four points. (**c**) Comparison of the distance from the modiolus to the lateral wall (i.e., Dist(M,LWi)) at four points. Point 1, upper basal turn; Point 2, middle turn; Point 3, lower apical turn; Point 4, upper apical turn; ns, no statistical significance; *, statistical significance.

As depicted in Fig. 4, modified ICPI-related metrics on the Cochlear view could be clearly determined at four fixed positions under a two-axis crossing the modiolus in the conventional CT with a high-resolution filter. Our observed values, including those for modified ICPI and their associated metrics, showed well-preserved normality, following a Gaussian distribution (Table S2). Furthermore, the average angular depth of insertion (i.e., the final position of the electrode tip) was 375.7 ± 14.8 (range 350.3–403.3), revealing very low standard deviation of the angular depth of insertion. Thus, the electrode configuration of the cohorts included in this study was hypothesized to recapitulate the schematic illustration (see Fig. 2a) and now we see that variability of position of slim modiolar electrodes along the array due to differences in cochlear shape and size is not significant.

Under the equation, the average modified ICPI of slim modiolar electrodes implanted by the round window approach, exclusively with the pull-back technique, was 0.44 ± 0.05, ranging from 0.34 to 0.54. The distance from the modiolus to the electrode remained unchanged between point 1 (i.e., electrode No.7-8), point 2 (i.e., electrode No. 12-13), and point 3 (i.e., electrode No. 16-17), while point 4 (i.e., electrode No. 20-21) showed a sudden significant decrease (*P* < 0.001 by one-way ANOVA and post-hoc Tukey test) (Fig. 4b). On the other hand, the distance from the modiolus to the lateral wall gradually decreased from point 1 to point 4 with statistical significance (Fig. 4c). Overall, ICPI(i) tended to increase from the upper basal turn (i.e., electrode No. 7-8) to the apical turn (i.e., electrode No. 20-21). That is, ICPI (i) at point 1 exhibited the lowest value due to the longest distance from the modiolus to the lateral wall, while relatively maintaining the distance from the modiolus to the electrode (Fig. 4a).

### Functional relevancy for our proposed CT measures

Intraoperative ECAP thresholds varied across electrode arrays, demonstrating that the ECAP thresholds tended to gradually increase from the apical to basal cochlear region (Fig. S3). By definition, ICPI values indicate the distance of each contact relative to the modiolus. This measurement is normalized by the electrode, being zero “0” the closest position to the modiolus and one “1” the closest position to the lateral wall^25^. Amongst four ICPI values, the ICPI at point1, which presented the lowest intracochlear position index (ICPI) value, displayed the lowest ECAP threshold (Fig. S4). In addition, the average ECAP threshold between point 1, point 2, and point 3 did not differ, suggesting that the modiolus-electrode distance presented in the current study correlates with the ECAP thresholds across electrode arrays (Fig. S4). The longer the modiolus-electrode distance, the higher the ECAP threshold. Resultantly, ICPI values presented in the current study likely show an inverse correlation with the ECAP thresholds at respective points, suggesting the effectiveness of our proposed measure of electrode array position on prediction of stimulability of the auditory nerve. Accordingly, functional relevancy justifies our CT measurement.

### Correlation of modified ICPI with spiral diameter

**Figure 5:**
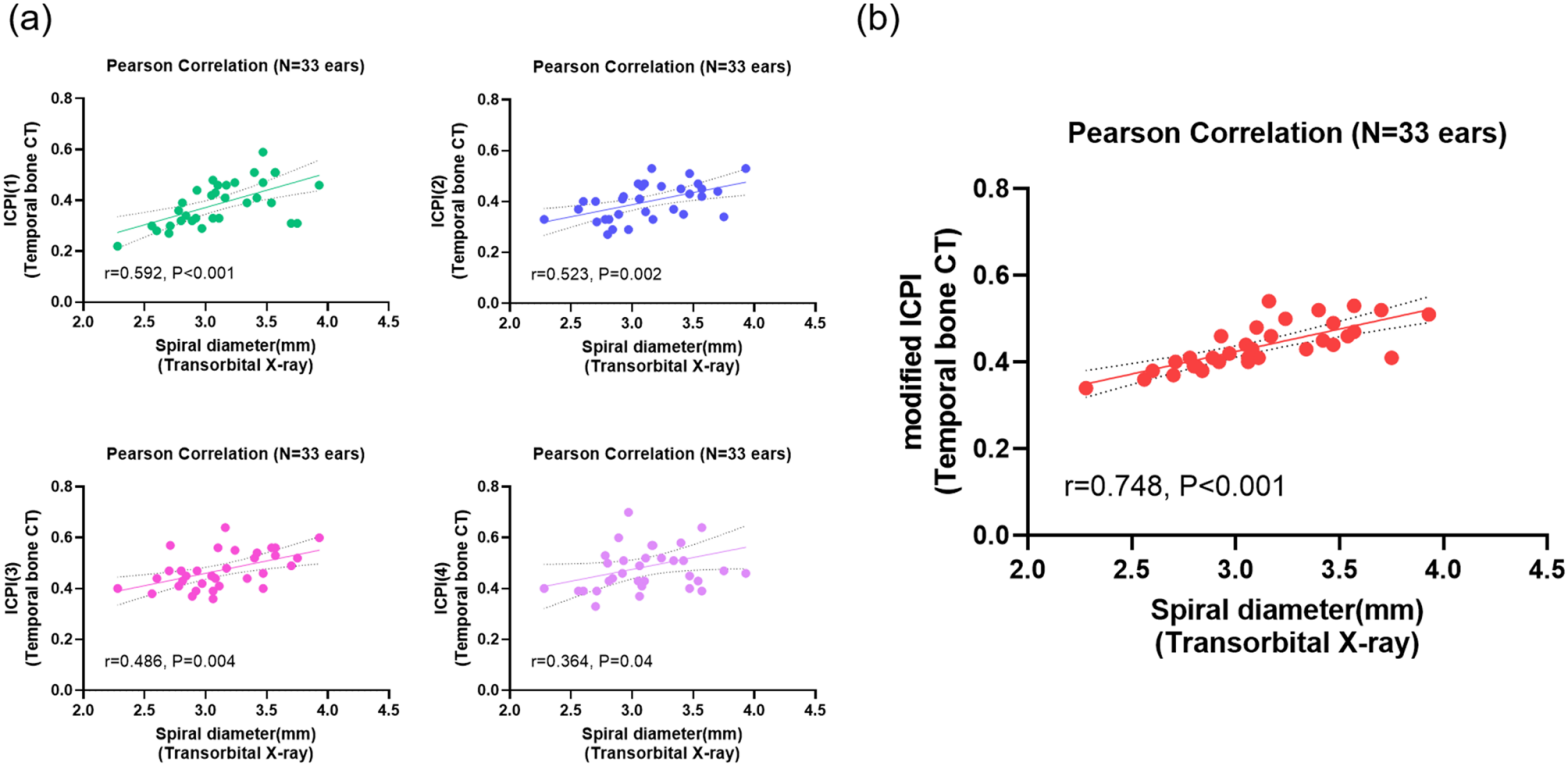
Correlation analyses of modified intracochlear position index (ICPI)-related metrics and X-ray-based spiral diameter. (**a**) Using Pearson correlation analyses, the X-ray-based spiral diameter was found to be correlated with ICPI(1) (r = 0.592, *P* < 0.001), ICPI(2) (r = 0.523, *P* = 0.002), ICPI(3) (r = 0.486, *P* = 0.004), and ICPI(4) (r = 0.364, *P* = 0.04), respectively. (**b**) Using Spearman correlation analyses, the modified ICPI was found to show much tighter correlation with the spiral diameter on the post-insertion radiograph (r = 0.748, *P* < 0.001) than did any of each ICPI at four points. The dotted line indicates the 95% confidence interval. *CT* computed tomography.

Pearson correlation analysis was performed to identify any correlation, beyond just association, between the modified ICPI or its metrics and previously proposed parameter of modiolar proximity, the spiral diameter, measured from a post-insertion radiograph during or after cochlear implantation. The mean spiral diameter was 3.12 ± 0.38 mm, exhibiting well-preserved normality that exactly followed a Gaussian distribution. A significant positive correlation was consistently observed between each ICPI value and the spiral diameter on the transorbital view (Fig. 5a). Amongst four ICPI values, the ICPI (1) measured on electrode No. 7-8, which presented the lowest value amongst metrics (see Fig. 4a), displayed the most significant correlation with the spiral diameter (r = 0.592, *P* < 0.001) relative to other ICPI values, likely indicating that modiolus-electrode distance at position 1 would most significantly contribute to spiral diameter.  Interestingly, the most significant correlation was obtained between average value of four ICPIs (modified ICPI) and spiral diameter (r = 0.748, *P* < 0.001) (Fig. 5b). This suggests that our current method of spiral diameter measurement using a post-insertion radiograph comprehensively reflects modiolus-electrode distances at point 1, 2, 3 and 4 rather than a specific point, despite being a two-dimensional evaluation. This underlines the obvious role of this indicator reflecting optimal modiolar proximity.

## Discussion

To the best of our knowledge, the present study is the first to come up with a reliable and simple parameter reflecting the modiolar proximity of slim modiolar electrodes based on conventional CT. Specifically, we adopted ultra-high kernel specification with high resolution index for conventional CT, finally achieving better image sharpness and reduced metal artifacts (partial volume artifacts). This improved image quality allowed accurate assessment of the intracochlear electrode position and elaborate measurement of modiolar proximity. The vendor-specific application of noise amplitude and texture modification technique in ultra-high kernel specification may decrease noise level and improve resolution. Indeed, conventional CT-based scalar position and distance from electrodes to the modiolus were comparable to the results of cone beam CT in certain circumstances^28^. Further, upon ultra-high kernel specification with high-resolution index, the enhancement of intracochlear visualization of the electrode array in conventional CT could justify the newly introduced indicator (i.e., modified ICPI) specialized for modiolar proximity evaluation. The modified ICPI measured from our cohort in conventional CT ranged from 0.34 to 0.54, which showed a normal distribution. Introduction of modified ICPI would make it possible to accurately and routinely evaluate modiolar proximity for slim modiolar electrodes in cochlear implantation centers where it was previously impossible due to lack of cone-beam CT. This would popularize analysing postoperative speech outcome with relation to modiolar proximity.

Recent studies have shown a diverse range of spiral configuration of slim modiolar electrodes due to intrinsic and extrinsic factors^4,7,36^, raising the importance of a de facto real-time imaging modality visualizing degree of modiolar proximity on the spot. A recent study highlighted that the cochlear duct length-based customized insertion technique might potentially ensure better modiolar proximity^20^, which would potentially lead to better speech outcomes. Specifically, short cochlear duct length subsequently leads to less modiolar proximity, which in turn renders some subjects with short cochlear duct length less amenable to this pull-back maneuver. If that is the case, readjustment of the electrode position by pulling the electrode array further back 1–2 mm more than in the conventional pull-back maneuver, called ‘extended pull-back maneuver’, likely leads to enhanced modiolar proximity, as evidenced by our recent study^20^. Coupled with reloadable design of the slim modiolar electrodes, simple X-ray imaging would serve as a great alternative. Previous studies have demonstrated the excellent performance of the post-insertion simple radiographs in the evaluation of the depth and angle of insertion of electrodes in intraoperative and postoperative settings^32,37^. What about evaluation of modiolar proximity?

Despite previous reports addressing the parameter of modiolar proximity (e.g., spiral diameter) based on the post-insertion simple radiograph, whether or not two-dimensional spiral diameter measured at a fixed position could comprehensively reflect three-dimensional spiral configuration of slim modiolar electrodes remained elusive. In this perspective, the present study merits strong attention since the modified ICPI comprehensively reflects electrode position at four points of the first cochlear turn, exhibited much tighter correlation with the spiral diameter on the post-insertion radiograph than did any of each ICPI at four points. This, in turn, suggests that the X-ray imaging-based spiral diameter would reflect the distance between the several contacts and the modiolus. Intraoperative use of simple radiographs to evaluate the degree of modiolar proximity, angular insertion depth and intracochlear positioning of electrodes under minimal exposure to radiation^38^ seems judicious, particularly in children. Taken together, our results reiterate the value of X-ray-based measurement of spiral diameter, which enables immediate visual feedback and changes in insertion technique during operation, thereby ensuring optimal modiolar proximity.

However, CT measurement should not be underestimated as just an interim approach, but we would better regard it as a valuable independent approach per se. Indeed, simple X-ray is not possible to indicate the degree of modiolar proximity of the electrodes, without being equipped with cone beam CT and calculation of ICPI through it. Importantly, each ICPI (i) could provide otologists with clinically significant information on intracochlear positioning of slim modiolar electrode, which is primarily related to the distance of each contact relative to the modiolus at specific points. Importantly, these values can be measured at a fixed position based on the two lines associated with the modiolus and the round window, thus permitting excellent reproducibility. Among them, ICPI (1) measured at junction of proximal basal and distal middle region (i.e., Electrode No. 7-8) showed the lowest value due to the longest distance from the modiolus to the lateral wall, while relatively maintaining the distance from the modiolus to the electrode. The enrolled subjects were exclusively implanted using the pull-back technique, enabling closer positioning of this slim modiolar electrode array to the modiolus. Recently, a human cadaveric temporal bone study revealed that the pull-back technique led to better modiolar proximity of the slim modiolar electrode array, especially in upper basal and middle turn of the cochlea^29^. Indeed, our measurement herein showed no difference of the distance among the electrode contacts from modiolus at point 1 (upper basal turn), 2 (middle turn), and 3 (lower apical turn). This enhanced modiolar proximity, especially at point 1 (upper basal turn) and 2 (middle turn), could be the result of the pull-back technique. Meanwhile, the distance between the modiolus and the lateral wall, which gradually decreased along the trajectory approaching the apical region, was consistent with the histological findings of the human cochlea^39^. Remarkably, we noticed that ICPI (1) with the lowest value also had the most tight correlation with X-ray imaging-based spiral diameter amongst the four ICPI values. This may indicate that changes in modiolar proximity in the upper basal cochlear region corresponding to ICPI (1) may be the most sensitive in influencing the spiral configuration of the electrode array. With a reduction of the electrode volume up to 75% similar to that of the current lateral wall electrodes^4^, the thinner and flexible properties of the slim modiolar electrode may make it more prone to variable spiral configuration in the upper basal cochlear region, compared with the middle and apical region. Further, the highest standard deviation of ICPI (1) indicating a wide range of degree of modiolar proximity, particularly in the upper basal region, may have contributed to the most significant correlation of it with the X-ray imaging-based spiral diameter.

As with introduction of significant novel findings in literature, the present study also has limitations that should be addressed in future studies. First, the small sample size and retrospective study design could be associated with weak statistical power, despite the fact that our observed values exhibited well-preserved normality, which followed a Gaussian distribution. Second, although previous studies substantiated that the electrode to modiolus distance based on conventional CT was significantly correlated with that based on a histological evaluation, the gold standard technique^28^, in vivo evaluation of the modified ICPI itself, was not validated histologically in our study. We therefore suggest that future studies should include large scale histological evaluation. Third, the structure of the cochlea is relatively small and irregular; therefore, it is difficult to designate the ROI when performing quantitative and qualitative assessments between standard and high-resolution filter specifications. As a result, the average ROI was relatively small and heterogeneous. Lastly, we cannot fully explain the impact of electrode position on speech performance at present, just postulating that improved modiolar proximity might lead to better speech perception outcomes in cochlear implant recipients. Specifically, Holden et al. proposed that a tightly wrapped array (i.e., wrapping factor) elicits significantly higher word recognition scores^1^. Furthermore, a prediction model has revealed that the average modiolus-to-electrode distance strongly correlated with speech perception scores for perimodiolar electrodes, including slim modiolar electrodes^13^.

## Conclusion

Introduction of conventional CT-based modified ICPI would make it possible to accurately and routinely evaluate modiolar proximity for slim modiolar electrodes in all cochlear implantation centers. Further, our results reiterate the value of X-ray-based measurement of spiral diameter which enables immediate visual feedback and changes in insertion technique intraoperatively, thereby ensuring optimal modiolar proximity. Additional pre-processing techniques, such as the metal artifact reduction and three-dimensional image fusion techniques, could further enhance the clinical significance of conventional CT-based measurement of several parameters in cochlear implantation.

## Supplementary information

Supplementary Table S1.

Supplementary Table S2.

Supplementary Figure S1.

Supplementary Figure S2.

Supplementary Figure S3.

Supplementary Figure S4.

## Data Availability

Data for all submitted results is available.
