# Supplementary material for: Simpler and effective radiological evaluations for modiolar proximity of a slim modiolar cochlear implant electrode
Source: Sci Rep. 2020 Oct 19;10:17714. doi: 10.1038/s41598-020-74738-x (PMC7573622; doi:10.1038/s41598-020-74738-x)
Supplement: Supplementary file 1 — Supplementary Table S1. [file 41598_2020_74738_MOESM1_ESM.pdf]

Simpler and effective radiological evaluations for modiolar proximity of a slim modiolar cochlear implant electrode

Sang-Yeon Lee, Jin Hee Han, Marge Carandang, Yun Jung Bae, Byung Yoon Choi

Supplementary Table 1. Genotype profiles of our cohort with causative variants

Subject	Gene	HGVS Nucleotide change	HGVS Protein change	Inheritance	Locus
1	*USH2A* [NM_206933.2] [NP_996816]	c.14835del	p.Val4946Trpfs*4	AR (compound heterozygote)	USH2A
		c.13112_13115del	p.Gln4371Argfs*19		
10	*ACTG1* [NM_001199954.1] [NP_001186883]	c.1013C>T	p.Ser338Leu	AD (heterozygote)	DFNA20
11	*MYO15A* [NM_016239.3] [NP_057323.3]	c.5504G>A	p.Arg1835His	AR (compound heterozygote)	DFNB3
		c.10245_10247del	p.Ser3417del		
14	*GJB2* [NM_004004.5] [NP_003995]	c.235del	p.Leu79Cysfs*3	AR (homozygote)	DFNB1A
18	*PAX3* [NM_181459.3] [NP_852124]	c.808C>T	p.Arg270Cys	AD (heterozygote)	WS
19	*SLC26A4* [NM_000441.2] [NP_000432]	c.1229C>T	p.Thr410Met	AR (compound heterozygote)	DFNB4
		c.2168A>G	p.His723Arg		
29	*SLC26A4* [NM_000441.2] [NP_000432]	c.2168A>G	p.His723Arg	AR (homozygote)	DFNB4
36	*MT-TL1* [NC_012920.1]	m.3243A>G	-	Maternal inheritance (heteroplasmy)	tRNA-leu (UUR) gene

Abbreviation: HGVS, human genome variation study; AR, autosomal recessive; AD, autosomal dominant
