# Supplementary material for: Simpler and effective radiological evaluations for modiolar proximity of a slim modiolar cochlear implant electrode
Source: Sci Rep. 2020 Oct 19;10:17714. doi: 10.1038/s41598-020-74738-x (PMC7573622; doi:10.1038/s41598-020-74738-x)
Supplement: Supplementary file 2 — Supplementary Table S2. [file 41598_2020_74738_MOESM2_ESM.pdf]

Simpler and effective radiological evaluations for modiolar proximity of a slim modiolar cochlear implant electrode

Sang-Yeon Lee, Jin Hee Han, Marge Carandang, Yun Jung Bae, Byung Yoon Choi

Supplementary Table 2. Distribution of modified ICPI and its related metrics

	ICPI (1)	ICPI (2)	ICPI (3)	ICPI (4)	modified ICPI
Average & Consistency					
Mean (SD)	0.39 (0.08)	0.40 (0.06)	0.47 (0.07)	0.49 (0.10)	0.44 (0.05)
range	0.22-0.59	0.27-0.53	0.36-0.64	0.33-0.84	0.34-0.54
Test for normal distribution					
Anderson-Darling test					
P-value	0.249	0.357	0.433	0.298	0.184
D'Agostino & Pearson test					
P-value	0.682	0.467	0.301	0.088	0.092
Shapiro-Wilk test					
P-value	0.474	0.387	0.460	0.253	0.124
Kolmogorov-Smirnov test					
P-value	>0.100	>0.100	>0.100	>0.100	0.063

Abbreviation: ICPI, intracochlear position index; SD, standard deviation
