# Supplementary material for: Simpler and effective radiological evaluations for modiolar proximity of a slim modiolar cochlear implant electrode
Source: Sci Rep. 2020 Oct 19;10:17714. doi: 10.1038/s41598-020-74738-x (PMC7573622; doi:10.1038/s41598-020-74738-x)
Supplement: Supplementary file 4 — Supplementary Figure S2. [file 41598_2020_74738_MOESM4_ESM.pdf]

Simpler and effective radiological evaluations for modiolar proximity of a slim modiolar cochlear implant electrode

Sang-Yeon Lee, Jin Hee Han, Marge Carandang, Yun Jung Bae, Byung Yoon Choi

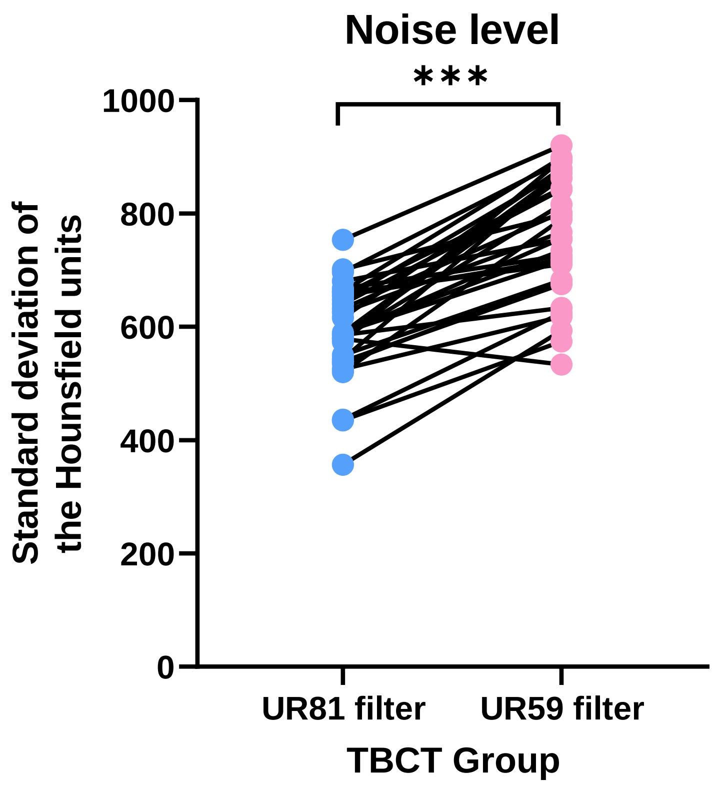

Supplementary Figure 2. Comparison of quantitative assessment in terms of noise level between two different resolution indices (UR81 filter vs. UR59 filter). TBCT, conventional temporal bone computed tomography. ***, statistical significance ($P < 0.001$) by paired t-test.
