# Supplementary material for: Simpler and effective radiological evaluations for modiolar proximity of a slim modiolar cochlear implant electrode
Source: Sci Rep. 2020 Oct 19;10:17714. doi: 10.1038/s41598-020-74738-x (PMC7573622; doi:10.1038/s41598-020-74738-x)
Supplement: Supplementary file 5 — Supplementary Figure S3. [file 41598_2020_74738_MOESM5_ESM.pdf]

Simpler and effective radiological evaluations for modiolar proximity of a slim modiolar cochlear implant electrode

Sang-Yeon Lee, Jin Hee Han, Marge Carandang, Yun Jung Bae, Byung Yoon Choi

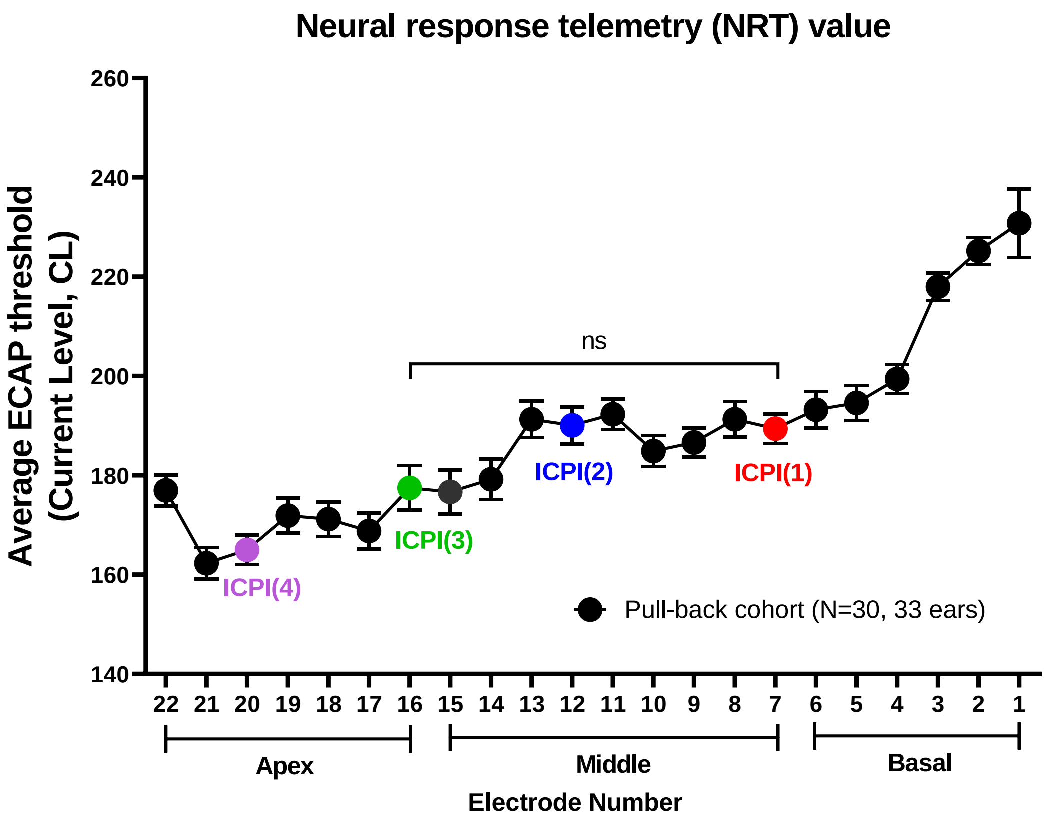

Supplementary Figure 3. The Intraoperative electrically evoked compound action potential (ECAP) thresholds. ECAP thresholds tended to gradually increase from the apical to basal cochlear region. The average ECAP threshold between point 1, point 2, and point 3 did not differ. Amongst four intracochlear position index (ICPI) values, the ICPI at point 4 displayed the lowest ECAP threshold. ns: no statistical significance.
