# Supplementary material for: Simpler and effective radiological evaluations for modiolar proximity of a slim modiolar cochlear implant electrode
Source: Sci Rep. 2020 Oct 19;10:17714. doi: 10.1038/s41598-020-74738-x (PMC7573622; doi:10.1038/s41598-020-74738-x)
Supplement: Supplementary file 6 — Supplementary Figure S4. [file 41598_2020_74738_MOESM6_ESM.pdf]

Simpler and effective radiological evaluations for modiolar proximity of a slim modiolar cochlear implant electrode

Sang-Yeon Lee, Jin Hee Han, Marge Carandang, Yun Jung Bae, Byung Yoon Choi

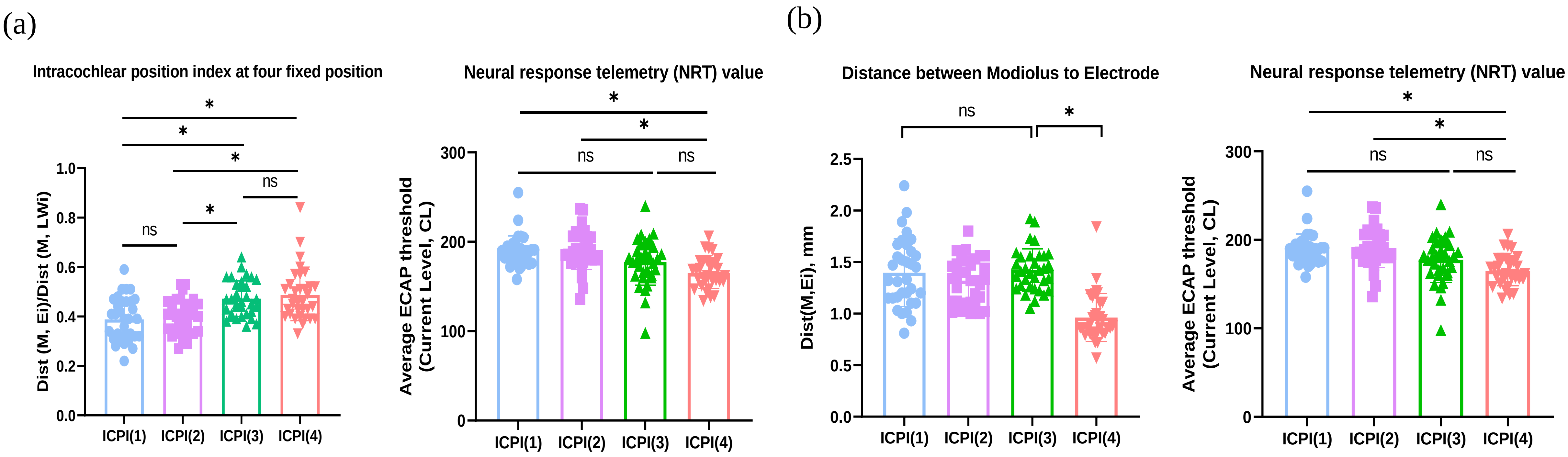

Supplementary Figure 4. Functional relevancy for our proposed CT measures. (a) Comparison of intracochlear position index (ICPI) values and average electrically evoked compound action potential (ECAP) thresholds at four fixed position, . (b) Comparison of electrode-modiolus distance and average ECAP at four fixed position. *, statistical significance; ns, no statistical significance
